# Co-infection of *Haemonchus contortus* and *Trichostrongylus* spp. among livestock in Malaysia as revealed by amplification and sequencing of the internal transcribed spacer II DNA region

**DOI:** 10.1186/1746-6148-10-38

**Published:** 2014-02-07

**Authors:** Tiong K Tan, Chandrawathani Panchadcharam, Van L Low, Soo C Lee, Romano Ngui, Reuben SK Sharma, Yvonne AL Lim

**Affiliations:** 1 Department of Parasitology, Faculty of Medicine, University of Malaya, 50603 Kuala Lumpur, Malaysia; 2 Veterinary Research Institute, 59, Jalan Sultan Azlan Shah, 31400 Ipoh, Perak, Malaysia; 3 Institute of Biological Sciences, Faculty of Science, University of Malaya, 50603 Kuala Lumpur, Malaysia; 4 Department of Veterinary Laboratory Diagnosis, Faculty of Veterinary Medicine, Universiti Putra Malaysia, 43400 UPM Serdang, Selangor, Malaysia

**Keywords:** Strongylid, *Haemonchus contortus*, *Trichostrongylus*, Infection rate, Livestock, Co-infection, Second internal transcribed spacer (ITS2) of ribosomal DNA

## Abstract

**Background:**

*Haemonchus contortus* and *Trichostrongylus* spp. are reported to be the most prevalent and highly pathogenic parasites in livestock, particularly in small ruminants. However, the routine conventional tool used in Malaysia could not differentiate the species accurately and therefore limiting the understanding of the co-infections between these two genera among livestock in Malaysia. This study is the first attempt to identify the strongylids of veterinary importance in Malaysia (i.e., *H. contortus* and *Trichostrongylus* spp.) by amplification and sequencing of the Internal Transcribed Spacer II DNA region.

**Results:**

Overall, 118 (cattle: 11 of 98 or 11.2%; deer: 4 of 70 or 5.7%; goats: 99 of 157 or 63.1%; swine: 4 of 91 or 4.4%) out of the 416 collected fecal samples were microscopy positive with strongylid infection. The PCR and sequencing results demonstrated that 93 samples (1 or 25.0% of deer; 92 or 92.9% of goats) contained *H. contortus*. In addition, *Trichostrongylus colubriformis* was observed in 75 (75.8% of 99) of strongylid infected goats and *Trichostrongylus axei* in 4 (4.0%) of 99 goats and 2 (50.0%) of 4 deer. Based on the molecular results, co-infection of *H. contortus* and *Trichostrongylus* spp. (*H. contortus* + *T. colubriformis* denoted as HTC; *H. contortus* + *T. axei* denoted as HTA) were only found in goats. Specifically, HTC co-infections have higher rate (71 or 45.2% of 157) compared to HTA co-infections (3 or 1.9% of 157).

**Conclusions:**

The present study is the first molecular identification of strongylid species among livestock in Malaysia which is essential towards a better knowledge of the epidemiology of gastro-intestinal parasitic infection among livestock in the country. Furthermore, a more comprehensive or nationwide molecular-based study on gastro-intestinal parasites in livestock should be carried out in the future, given that molecular tools could assist in improving diagnosis of veterinary parasitology in Malaysia due to its high sensitivity and accuracy.

## Background

Nematode parasites commonly known as strongylids belonging to the order Strongylida and superfamily Trichostrongyloidea significantly affect the health of livestock [1]. Among these strongylid species, *Haemonchus contortus* and *Trichostrongylus* spp. are reported to be the most prevalent and highly pathogenic in livestock, particularly in small ruminants. It is indisputable that *H. contortus* is the most notorious parasite in livestock (i.e., ruminants) due to its biotic potential and blood sucking ability [2]. *Haemonchus contortus* infection (i.e., haemonchosis) may exhibit clinical signs such as anemia, followed by lack of appetite, lethargy, loss of weight, dehydration, oedema and death as a consequence of the disease [2-5]. As compared to *H. contortus*, *Trichostrongylus* infection may show milder clinical signs, which may result in inappetence, weight loss, poor body condition, emaciation, diarrhea, hypoproteinaemia and death in the case of heavy infection, particularly in malnourished animals [5,6].

In animal treatment management, species identification of strongylid is often deemed unnecessary; given that drug treatment is usually similar for the different species. Nonetheless, strongylid species identification is crucial in obtaining a greater understanding of the epidemiology, population biology and anthelmintic treatment efficacy, all of which are essential factors for formulating effective parasite control strategies. It is important to emphasize that this information is rarely obtained from conventional diagnostic technique. Strongylid species can only be successfully identified via advanced tools such as molecular techniques. It is important then to know this fact as it is possible that an individual animal could be susceptible to more than one strongylid species when several species are circulating in a farm pasture [7,8]. The occurrence of mixed infections may pose a serious problem as they could aggravate the health consequences of the infected animal.

In Malaysia, detection of ova is routinely performed by a floatation principle and observation under a light microscope in veterinary diagnostic laboratories, namely, universities and government agencies (i.e., Department of Veterinary Services or DVS, Malaysia). Although this technique enables a wide range of parasite detection, information of genus and species cannot be easily deciphered. Given that each genus of strongylid has a certain range of egg sizes, the overlapping sizes make it more challenging to pinpoint its genus especially for inexperienced staff. Although fecal culture is another technique for strongylid identification by defining the specific genus characteristics at larval stage, this method is unfortunately time-consuming and requires technical expertise. Furthermore, the accuracy of identification may be questionable and it is impossible to identify the strongylid up to species level.

The utilization of molecular tools such as PCR and DNA sequencing has enabled the accurate identification of parasite species [9]. These advanced techniques are highly sensitive, providing highly accurate identification of strongylids up to species level. Starting from 1990, the Internal Transcribed Spacer (ITS) of nuclear ribosomal DNA (i.e., Second Internal Transcribed Spacer or ITS2) has been developed as a reliable genetic marker in strongylid species identification [9-13] due to its high interspecific sequence divergence and intraspecific sequence homogeneity [14,15]. Among these studies, Bott et al. [12] developed a real time-PCR coupled with melting curve analysis based on the ITS2 of ribosomal DNA for the improvement in veterinary parasitology diagnosis on seven common strongylid parasites, namely *H. contortus*, *Trichostrongylus* spp., *Teladorsagia circumcincta*, *Cooperia oncophora*, *Chabertia ovina*, *Oesophagostomum columbianum* and *Oesophagostomum venolosum* in small ruminants.

In the present study, species specific primers from Bott et al. [12] were applied to amplify ITS2 DNA region of *H. contortus* and *Trichostrongylus* spp. from microscopy positive fecal samples of Malaysian livestock. This study is the first attempt to accurately identify the Stronglyes of veterinary importance in Malaysia (i.e., *H. contortus* and *Trichostrongylus* spp.) by molecular methods. The application of advanced molecular tools in determining the specific identity of strongylid species will provide complementary evidence to the microscopy detection of eggs and larvae.

## Results

A total of 416 rectal fecal samples from four types of livestock (i.e., 98 cattle; 70 deer, 157 goats and 91 swine) were examined (Table 1). Among the examined samples, 118 (11 or 11.2% of cattle; 4 or 5.7% of deer; 99 or 63.1% of goats; 4 or 4.4% of swine) were microscopically positive for strongylid parasites and these parasites were subsequently subjected to molecular identification of *H.contortus* and *Trichostrongylus* spp.

**Table 1:** Number of strongylid positive samples by microscopy and PCR from different type of livestock

**Livestock**	**No. examined**	**Microscopy positive**		**PCR positive**			
				***Haemonchus contortus***		***Trichostrongylus *** **spp.**	
		**No.**	**%**	**No.**	**%**	**No.**	**%**
Cattle	98	11	11.2	0	0	0	0
Deer	70	4	5.7	1	25.0	2	50.0
Goat	157	99	63.1	93	93.9	79	79.8
Swine	91	4	4.4	0	0	0	0
**Total**	**416**	**118**	**28.4**	**94**	**79.7**	**81**	**68.6**

ITS2 DNA region of *H. contortus* was amplified in 94 (79.7% of 118) individuals, consisting of 93 isolates from goats (93 of 99 or 93.9%) and one from deer (1 of 4 or 25%) (Table 1). Of these, 92 amplicons were successfully sequenced and represented by two distinct sequence types [GenBank accession numbers KF204571 and KF204572]. Neighbour-Joining analysis revealed that both sequences were clustered with *H. contortus* sequences available from GenBank (99-100% similarity) and apparently differed from its closely related species *H. placei*. As for *Trichostrongylus* spp. detection, a total 81 amplicons were amplified, comprising 79 goats (79.8% of 99) and two deer (50.0% of 4). Of these, all amplicons were successfully sequenced revealing five sequence types [GenBank accession numbers KF204573 to KF204577]. Neighbor-Joining analysis of these five sequences demonstrated the occurrence of two *Trichostrongylus* species infection in the studied individuals. Among the representative sequences, KF204573 and KF204574 belonged to *Trichostrongylus axei* (100% similarity), while the remaining sequences (i.e., KF204575, KF204576 and KF204577) were identified as *Trichostrongylus colubriformis* with 98-100% similarity to the published sequences in GenBank (Table 2). In goats, *T. colubriformis* (75 of 79 or 94.9%) was more predominant than *T. axei* (4 of 79 or 5.1%). In contrast, only *T. axei* (2 of 2 or 100%) was detected in deer. Overall, in goats, the infection rate of *H. contortus* was 58.6% (92 of 157) followed by *T. colubriformis* (47.8% or 75 of 157) and *T. axei* (2.5% or 4 of 157). With regards to deer, *T. axei* (2.9% or 2 of 70) reported higher infection rate than *H. contortus* (1.4% or 1 of 70).

**Table 2:** ***Haemonchus contortus*, *Trichostrongylus colubriformis *** **and *Trichostrongylus axei *** **in livestock fecal samples (microscopically strongylid positive) determined by DNA sequencing and Neighbor-Joining analysis according to type of livestock**

**Livestock**	**PCR positive (primer HAE and NC2)**	***H. contortus*** *		**PCR positive (primer TRI and NC2)**	***Trichostrongylus *** **spp.**			
					***T. colubriformis*** *		***T. axei*** *	
		**No.**	**%**		**No.**	**%**	**No.**	**%**
Deer	1	1	100.0	2	0	0	2	100.0
Goat	93	92	98.9	79	75	94.9	4	5.1
**Total**	**94**	**93**	**98.9**	**81**	**75**	**92.6**	**6**	**7.4**

*Species identity confirmed by Neighbour-Joining Analysis.

With regards to single parasitic infection, in goats, single *H. contortus* infection (17 of 157 or 10.8%) exhibited the highest infection rate, followed by *T. colubriformis* (4 of 157 or 2.5%) and *T. axei* (1 of 157 or 0.6%). Moreover, mono-parasitism was also detected in deer. Among the strongylid positive individuals, co-infections of both strongylid species (HTC denoting *H. contortus*+*T. colubriformis* infections; HTA denoting *H. contortus*+*T. axei* infections), HTC and HTA infections were only observed in goats, with HTC infections (71 of 157 or 45.2%) being more predominant than HTA (3 of 157 or 1.9%) (Table 3). As for deer, no poly-parasitism (double infections) was found in the present study.

**Table 3:** **Single infection and co-infection of *Haemonchus contortus*, *Trichostrongylus colubriformis *** **and *Trichostrongylus axei *** **in deer and goats**

**Parasitism**	**Deer**		**Goat**	
	**No.**	**%**	**No.**	**%**
**Single infection**				
*H. contortus*	1	1.4	17	10.8
*T. colubriformis*	0	0	4	2.5
*T. axei*	2	2.9	1	0.6
**Co-infection**				
*H.contortus*+*T. colubriformis*	0	0	71	45.2
*H. contortus*+*T. axei*	0	0	3	1.9

## Discussion

In Malaysia, the molecular detection of parasites of veterinary importance in livestock such as *Giardia*[16], *Cryptosporidium*[17,18], *Neospora caninum*[19] have been reported. However, there is a conspicuous lack of molecular data focusing on strongylid parasites, the most pathogenic group of GIP to livestock in Malaysia. In the present study, the most common strongylid parasite, *H. contortus* infection was found in 22.4% (93 of 416) of studied animals, comprising 92 goats (58.6% of 157) and one deer (1.4% of 70). A number of drug resistance studies in Malaysia have indicated that *H. contortus* remains the most widespread strongylid species (73–97%) in small ruminants (i.e., goats and sheep) [20-23]. These studies have indirectly acknowledged the preponderance of *H. contortus* infection in Malaysia and the current study further confirms this notion. Likewise, the predominance of this parasite species in goats has been reported worldwide. In comparison with previous studies, the prevalence of strongylids noted in this study was much lower than Kenya (90%) [24], Zimbabwe (88-97%) [25] and Brazil (96.9%) [26].

In contrast, *H. contortus* was less common among the studied domesticated deer. Similar findings were also noted in the red deer in Stelvio National Park, one of the main protected areas of north-eastern Italy (1.3%) [27] and roe deer in the northwest of Iberian Peninsula, Spain (1.4%) [28]. The results indicated this species might not be a major threat to the wellness of deer [27]. Nonetheless, this finding must not be generalized and a more comprehensive study in the country should be conducted.

*Trichostrongylus* infection was also observed in the present study. Although *Trichostrongylus* is less significant to livestock compared to *H. contortus*, its impact on livestock cannot be underestimated [5,29]. In Malaysia, a series of drug resistance studies reported that *Trichostrongylus* (5–26%) was the second predominant strongylid parasite species among livestock after *H. contortus* (73–97%) [20-23]. In recent years, there is an increasing trend of *Trichostrongylus* infection in small ruminants (personal communication, Veterinary Research Institute, Malaysia). Not surprisingly, more than half of the strongylid infected goats and deer in the present study were positive for *Trichostrongylus*. Among the *Trichostrongylus* species, *T. colubriformis* was the most common species in goats. The infection of small ruminants with this species appears to be common with a wide spectrum of prevalence rates, as high as 78–100% in Nigeria [30,31], more than 90% in France [32] and as low as 9.8% in Iran [33]. Apart from small ruminants, *T. colubriformis* infection has also been reported in other livestock including cattle [5,29,34]. However, none of the *Trichostrongylus* species was detected among cattle samples in the present study.

As for *T. axei*, its predomination in temperate zones around the world have been pointed out [35], such as Nigeria (69.2%) [31], Australia (overall more than 90%) [36] and Zimbabwe (88–97%) [25]. However, in Malaysia (a tropical country), there is only one study reporting that *T. axei* was the most common strongylid parasite as observed from post-mortem examination of small ruminants [37]. This is in contrast with the findings of the present study, where a very low frequency of *T. axei* was demonstrated. It is important to point out that the current status of *T. axei* in Malaysia remains unknown and therefore pinpoints the need for additional concerted research efforts in future.

Based on the results, none of the cattle and swine samples were positive for *H. contortus* and *Trichostrongylus* spp. Generally, *Haemonchus placei*, *Cooperia pectinata*, *Cooperia punctate* and *Ostertagia* spp. were the dominant strongylid parasites in cattle, notably in Kenya and Netherlands [38-40]. Attempts to amplify these species using the primer sets of Gasser et al. [9] were made but no positive samples found in the present study (unpublished data). Interestingly, the canine specific hookworm species (*Ancylostoma caninum*) was detected in one of the cattle sample in the present study as confirmed by DNA sequencing (data not shown). There is a high possibility of the cattle being a mechanical transporter. However, the actual factor(s) that contribute to this rare case need to be further investigated.

With regards to swine, absence of *H. contortus, T. axei* and *T. colubriformis* was observed among current studied swine samples. Although the occasional existence of *T. axei* has been described [5,29], its prevalence remained low (<5%) [41] and less significant to swine. As compared to *T. axei*, *T. colubriformis* is more common in swine where the natural incidental infections of *T. colubriformis* have been reported in Hungary, Australia, Russia and United Kingdom [42]. However, little attention has been paid to gastrointestinal parasitic infections in swine in Malaysia. It is crucial that a comprehensive coverage of the current status of parasitic infections in swine populations in Malaysia is conducted in future.

Even though there have been a number of publications stating co-infections of strongylid in animals [29,30], limited scientific reports demonstrated the real situation within host especially Malaysian livestock. The present study demonstrated that co-infections with *H. contortus* and *T. colubriformis* were predominant in goats. Fakae and Chiejina [30] have reported the co-occurrence of these strongylids (i.e., *H. contortus* and *T. colubriformis*) in goats ranging from 90% to 100%, which was significantly higher than the present study. With regards to co-existence of *H. contortus* and *T. axei* in goats, only a low number of strongylid positive samples were observed. Given the limited information currently available regarding the Malaysian *T. axei*, this result is crucial in filling the gap of knowledge of parasitic infection among ruminants in Malaysia.

Co-infection between *H. contortus* or *T. colubriformis* with other gastrointestinal parasites in small ruminants has been reported. Among these two strongylid species, *T. colubriformis* commonly co-occurred with other GIP to produce a more severe impact compared to single infection to the host. For example, *Ostertagia circumcincta* co-infected with *T. colubriformis* has been shown to significantly reduce wool growth in lambs (up to 66%) [43] while *Eimeria* spp. and *T. colubriformis* infections resulted in enteritis [29]. As for *H. contortus*, severe impacts (i.e., inappetence, severe scouring, and reduction in live body weight and death) have also been demonstrated during co-infection with *Eimeria* spp. [44].

With regards to co-infection of *H. contortus* or *T. colubriformis*, a series of studies reported the significant association between milk production and co-occurrence of these two strongylid species in dairy goats, which caused the reduction in milk yields (13.0-25.1%) in goats with highest milk production at the initial stage of the study [45]. In addition, Chartier and Hoste [46] found that repeated exposure to the mixture of *H. contortus* and *T. colubriformis* caused goats with high milk production to suffer more severe pathophysiological disturbances (increase in pepsinogen concentration and decrease in inorganic phosphate concentrations) and severe depression in milk yields (-8 – -35%). Given that the severe pathophysiology and productivity attributable to the co-infection of gastrointestinal parasites, the current results suggest the strongylids co-infected individuals might also suffer with similar impact of this phenomenon. Therefore, there is now an urgent need to investigate the co-infection status of strongylid parasites among Malaysian livestock for better treatment management.

## Conclusions

In summary, the present study is the first molecular identification of strongylid among Malaysian livestock which is essential for an in-depth understanding of the epidemiology of gastro-intestinal parasitic infection status in Malaysia. The findings of this study revealed the high infection rate of *H. contortus* in goats followed *by T. colubriformis* and *T. axei* with the co-existence of *H. contortus* and *Trichostrongylus* spp. infections in goats. Indeed, a comprehensive research such as nationwide investigation of GIP prevalence among livestock by the application of genotyping tools should be carried out in the near future. In addition, the authors would like to propose that the Malaysian government via its agency (i.e., Department of Veterinary Services) and research institute (Veterinary Research Institute) utilize the molecular screening tools for strongylid species identification. The accurate data will be very useful in some area such as the mapping in Geographical Information System (GIS) to determine the infection status, prevalence, distribution of strongylid parasites among the livestock in Malaysia. Also, to evaluate the future trends of strongylid infections among livestock in order to formulate more effective disease control programmes and worm management in Malaysia.

## Methods

### Ethical consideration

The study protocol was approved by the Ethics Committee of the University Malaya Medical Center, Malaysia (MEC Ref. No. 896.36). Permission for the study to be conducted on animal farms was obtained from owners prior to sample collection.

### Scatological procedure and fecal sample collection

The scatological survey was carried out in five farms located in three states in Malaysia, namely Selangor (Farm A in Serdang district), Perak (Farm B in Kuala Kangsar district; and Farm D in Batang Padang district) and Sarawak (Farm C and Farm E in Bau district). Among the farms studied, Farm A and Farm B reared more than two species of livestock (i.e., cattle, deer and goats). These animals are reared separately and speculated to have the lowest possibility of cross-GIP transmission. Goats were raised in Farms A, B and C, whereas, swine were raised in Farms D and E. The swine were raised under intensive farming while goats were kept in semi intensive management whereby they grazed in pastures during the day and housed in sheds at night. On the other hand, cattle and deer at farms A and B were managed extensively whereby they were allowed to graze in the fields or pastures. The age of the livestock ranged from 3 months to 3.5 years -in goats, 5 months to 9 years in cattle, 6 to 8 months in swine, and an average of 2 years in deer. All the studied farms were routinely monitored by the Department of Veterinary Services, Malaysia.

Fecal samples were collected per rectum from the animals studied. The fecal samples were tightly sealed in plastic bags and stored at 2°C to 8°C immediately after collection. Samples were transferred to laboratory and stored at −20°C until further analysis. The collected samples were processed by the formalin-ether concentration technique [47], followed by microscopic screening aided by Lugol’s iodine stain. The examination was performed using the 10X objective of a compound microscope for detection of GIP ova. The samples which were microscopically positive for strongylid eggs were further characterized by using genotyping tools.

### DNA isolation

The samples that were microscopically positive for strongylids were subjected to DNA extraction by using the QIAamp® DNA Stool Mini Kit (Qiagen, Germany) according to the manufacturer’s prescribed protocol. Approximately 200 mg of fecal sample was used for DNA extraction. Firm feces (i.e., goat and deer fecal pellets) were mechanically homogenized prior to DNA isolation following the manufacturer’s instructions. An additional step was taken during stool lysis; whereby silica beads were added to the fecal samples in a 2 ml centrifuge tube with 1.4 ml ASL buffer, followed by 10 minutes of horizontal vortexing with a vortex adapter (catalog no. 1300-V1; MO BIO Laboratories, Carlsbad, CA) before proceeding to incubation at 70°C for 10 minutes. A concentrated 50 μl of DNA was eluted and subsequently stored at −20°C prior to molecular genotyping of strongylid species.

### PCR amplification

A single step PCR was conducted to amplify the region of the second internal transcribed spacer (ITS2) of nuclear ribosomal DNA (rDNA) of the strongylid species (i.e., *H. contortus* and *Trichostrongylus* sp.). The amplifications were aided by two different pairs of primers in separate reactions and each reaction consisted of a species specific forward primer and a universal reverse primer for strongylid species. As for *H. contortus*, a product of 265 bp was amplified using the forward primer HAE (5′-CAA ATG GCA TTT GTC TTT TAG-3′) and the reverse primer NC2 (5′-TTA GTT TCT TTT CCT CCG CT-3′) [12]; as for *Trichostrongylus* spp., a product of 267-268 bp was amplified by the forward primer TRI (5′-TCG AAT GGT CAT TGT CAA-3′) and the reverse primer NC2 [12]. Precautions were taken to prevent contamination at every step of the procedure [i.e., PCR preparation was conducted in laminar flow cabinet, exposure of ultraviolet radiation (UV) on apparatus (micropipettes) and consumables (glove, micropipette tips, 1.5 ml and 200 μl tubes), separate rooms for DNA extraction and PCR etc.]. Each PCR was performed in a 50 μl reaction containing 10× PCR buffer, 2.5 mM dNTPs, 25 mM MgCl_2_, 10 pmol of each forward and reverse primer, 5 units of *Taq* polymerase and 6 μl of DNA template [48]. Negative (without DNA; replaced by nuclease free water) and positives (with DNA template of *H. contortus* and *Trichostrongylus* sp.) were also included in each PCR run. The PCR was carried out in the Bio-rad MyCyler™ Thermal Cycler Serial Number: 580BR 7200 (CA, USA). The cycling programme included 94°C for 5 min (initial denaturation), followed by 35 cycles of 94°C for 30 s (denaturation), 55°C for 30 s (annealing), 72°C for 30 s (extension), and a final extension at 72°C for 7 min [12].

### Sequence analysis and strongylid species identification

All the PCR amplified fragments were purified by QIAquick PCR Purification Kit (QIAGEN, Germany) according to manufacturer’s prescribed protocol prior to DNA sequencing. The purified PCR products were subjected to bidirectional DNA sequencing using the ABI PRISM 1 BigDyeTM Terminator v3.0 Ready Reaction Cycle Sequence Kit (Applied Biosystems, USA) in a 3700 DNA Analyzer (Applied Biosystems, USA). The obtained sequence chromatograms were viewed using Sequence Scanner 1.0 (Applied Biosystems, Foster City, CA). The sequence data were analyzed and preliminary aligned with the published reference sequences of *H. contortus* (KF36428-KF36432, HQ683710-HQ683715, FN432335-FN432336, JN12897-JN12898, JQ342246-JQ342247, X78803), *H. placei* (KF364623-KF364627, JN128895-JN128896, JQ342248-JQ342249, X78812) as presented in Jabbar et al. [49]; *T. axei* (KC998724-KC998727, AY439026) and *T. colubriformis* (AB503241-AB503243, AB503246, AB503250, AB503252, HQ844229) using BioEdit 7.0.9.0. [50]. The species identity was confirmed by Neighbor-Joining analysis using MEGA4 [51]. The Neighbour-Joining bootstrap values were estimated using 1000 replicates with Kimura’s two-parameter model of substitution (K2P distance) evolution model.

## Competing interests

The authors declare that they have no competing interests.

## Authors’ contributions

TKT research design, conduct research, data analysis, manuscript preparation; CP manuscript revision; VLL conduct research, manuscript revision; SCL conduct research; RN conduct research; RSKS research design, manuscript revision; YALL project leader, research design, manuscript revision. All authors read and approved the final manuscript.
